# Microfiber Interferometric Sensor for Ultrasound Detection

**DOI:** 10.3390/s26051739

**Published:** 2026-03-09

**Authors:** Xiuxin Wang, Jiwen Zhou, Shuojian Xiong Zheng, Zihao Wang, Bowen Tang, Hongzhong Li

**Affiliations:** 1 Medical Electronics and Information Technology Engineering Research Center, Chongqing Engineering Laboratory of Digital Medical Equipment and System, Chongqing University of Posts and Telecommunications, Chongqing 400065, China; 2 Chongqing Construction Automotive Systems Co., Ltd., Chongqing 404100, China; 3 School of Civil Engineering, Guangdong Communication Polytechnic, Guangzhou 510650, China

**Keywords:** ultrasonic detection, imaging and sensing, microfiber interferometer

## Abstract

By setting up ultrasonic fields in solid and liquid environments, the propagation characteristics of ultrasonic waves were investigated, and a sensing experiment device with related physical field settings was constructed. A comparison of results between multi-mode microfiber and single-mode fiber interferometric sensors found that the multi-mode microfiber maintains the original ultrasonic waveform output and has much higher sensitivity than the single-mode fiber sensor. The sensor in the paper had a detection limit of approximately 540 Pa and a bandwidth of approximately 5 MHz. The photoacoustic experiment with the microfiber ultrasound sensor had the highest resolution, which is about 10 times that of a single-mode fiber sensor. In summary, the multi-mode microfiber interferometric sensor was applied to ultrasonic detection.

## 1. Introduction

Ultrasonic detection technology, as a non-destructive and highly penetrating diagnostic method [1,2,3], has been widely employed in critical domains such as biomedical imaging, industrial non-destructive testing, and structural health monitoring. Traditional ultrasonic detection primarily relies on piezoelectric sensors; although their technology has matured, it is constrained by the physical characteristics of the sensors themselves, and there remain several insurmountable bottlenecks. For instance, piezoelectric elements are highly sensitive to electromagnetic interference, and there exists a strong coupling between their sensitivity and size—reducing the size of the element to achieve high spatial resolution often results in a significant decrease in detection sensitivity, which is particularly pronounced in high-frequency ultrasonic imaging. Furthermore, the acoustic impedance of piezoelectric ceramic materials is poorly matched with biological tissues or water media, necessitating complex matching layer designs, which limit their application in complex environments [4].

In response to these issues, optical fiber ultrasonic sensors, with their compact size, robust anti-electromagnetic interference capability [5], and excellent biocompatibility, have emerged as a highly promising alternative. Among the various optical fiber sensing structures [6], sensors based on Fabry–Perot interferometers (FPI) are favored for their compact design and adjustable sensitivity. Particularly, FPI sensors based on polymer membrane structures can achieve micron-scale dimensions while providing superior acoustic response [7], offering new technological avenues for endoscopic imaging and minimally invasive diagnostics [8]. However, conventional interferometric sensors still face physical limitations in sensitivity enhancement [9], often requiring expensive tunable lasers or complex demodulation systems to maintain the stability of the operating point, which increases the system’s complexity and cost.

To overcome the sensitivity bottleneck, sensing technology based on micro/nano optical fibers has garnered substantial attention in recent years [10]. Unlike standard single-mode fibers, micro/nano fibers reduce the core diameter through physical tapering, allowing the optical field to extend into the external medium of the fiber core in the form of evanescent waves. This extensive evanescent field characteristic significantly enhances the interaction between the optical field and the external acoustic field—when ultrasound acts on the fiber [11], the refractive index modulation and geometric deformation induced by sound pressure can be more effectively coupled to the optical signal through the evanescent field [12,13], thereby markedly improving the sensing sensitivity [14].

In this context, this paper presents a Fabry–Perot interferometric ultrasonic sensor based on micro/nano fiber gratings. In this configuration, the fiber Bragg grating (FBG) serves as a crucial element. FBG is a “wavelength-selective mirror” formed by periodic refractive index modulation within the fiber core. In this design, the grating, in conjunction with the fiber end face, forms a high-finesse F-P cavity, achieving high reflectivity at specific wavelengths and thereby constructing a stable interference cavity. Additionally, the large evanescent field effect of micro/nano fibers enables sensitive detection of acoustic signals.

Notably, existing research predominantly focuses on the fabrication and performance evaluation of the sensors themselves, whereas there is a relative scarcity of studies on the acoustic physical field model of ultrasonic wave propagation in various media (solid/liquid) and their interaction with optical fibers. This paper not only experimentally verifies the high sensitivity and low noise characteristics of the minimum detectable sound pressure of the sensor but also innovatively constructs an acoustic physical field model, simulating the propagation characteristics of ultrasonic waves in solid and liquid environments and their coupling mechanism with the sensor. The high consistency between experimental and simulation results validates the superior performance and potential applications of the micro/nano fiber FPI sensor in the field of ultrasonic detection.

## 2. Research on Pattern Interference in Multi-Mode Microfibers

Currently, optical fiber transmission has replaced traditional cable transmission in the communication field due to its low production cost, low transmission loss, high channel capacity [15], corrosion resistance, and electromagnetic interference immunity. Optical fiber sensing technology [16] has been in development since the 1970s, continuously demonstrating immense potential and a broad range of application prospects. Due to its unique advantages, optical fiber sensing technology has gained extensive and in-depth research and application in various fields [17], becoming a leader in the sensor technology domain.

Optical fibers were initially used as media for transmitting light waves and images. With the production of low-loss optical fibers, their application fields have greatly expanded, especially in communication technology, where optical fibers have become critical tools for long-distance information transmission. However, the functions of optical fibers are not limited to this; they can also be used as sensing elements. When light waves propagate through optical fibers, their characteristic parameters, such as amplitude, phase, polarization state, and wavelength, can vary due to external factors like temperature, pressure, electric fields, and rotation. This property allows optical fibers to be used as sensing elements, enabling precise detection of various physical quantities.

### 2.1. Characteristic Analysis

The COMSOL study is used to model a single-mode microfiber. The detailed modeling process is as follows: clarify the problem type and define parameters, draw the geometric model, followed by specifying material properties for each solving domain in the model, and then define loads and boundary conditions. Once the solution is complete, extract the obtained data and report the results. If needed, the model can also be modified and optimized based on the results.

**Figure 1:**
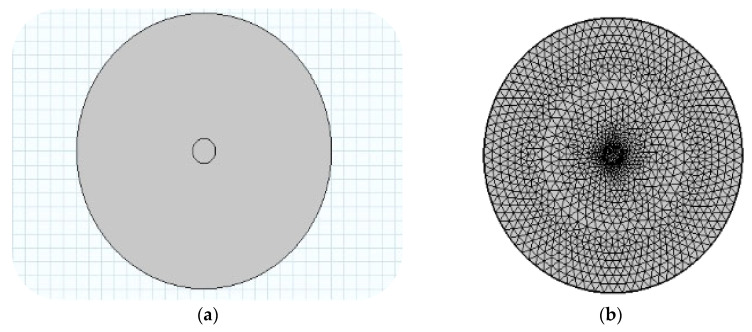
(**a**,**b**) Geometric model construction and mesh dissection of single-mode microfiber.

Initially, a two-dimensional cross-section diagram of the single-mode microfiber is designed, with the core diameter set to 0.3 μm and the cladding medium set to 25 μm. The wavelength of 1550 nm is specifically chosen as the incident wave, as it is widely used in fiber optic communication, including low attenuation, high bandwidth, and long-distance transmission capability. To achieve a more precise calculation of the optical field distribution, a free triangular mesh is chosen, using a predefined refinement method from the system. The method sets the maximum element size to 0.333 μm, the minimum element size to 0.00113 μm, the maximum element growth rate to 1.25, the curvature factor to 0.25, and the resolution of narrow regions to 1, see Figure 1.

The cladding medium is set to air, with a refractive index of 1, as the tapered cladding can be regarded as the surrounding environment. The core material is chosen as silica, which has a high refractive index, allowing light signals to propagate rapidly and effectively remain confined within the core. Additionally, silica offers good mechanical strength and chemical stability, ensuring the fiber maintains stable performance in various complex environments. The Sellmeier dispersion equation [18] is as follows (*λ* represents the wavelength of light, and *n* represents the refractive index):

(1) $$n^{2}-1=\frac{0.6961663\lambda^{2}}{\lambda^{2}-(0.0684043{)}^{2}}+\frac{0.4079426\lambda^{2}}{\lambda^{2}-(0.1162414{)}^{2}}+\frac{0.8974794\lambda^{2}}{\lambda^{2}-(9.896161{)}^{2}}$$

To further explore the transmission mode characteristics of microfibers with different diameters, a detailed mode analysis was conducted on fibers with diameters of 0.3 μm, 0.8 μm, 1.3 μm, and 1.8 μm, resulting in the calculation of the 2D light field distribution of the HE_11_ mode. By comparing the 2D light field distributions at these four different diameters, it can be observed that as the fiber diameter increases, the evanescent field on the surface of the microfiber gradually weakens [19]. This indicates that more light field energy is effectively confined within the core for transmission. Specifically, when the diameter of the microfiber is 0.3 μm, since its diameter is much smaller than the wavelength of the incident light, most of the light field energy in the HE_11_ mode disperses into the surrounding air medium in a strong evanescent field form. However, when the fiber diameter increases to 1.8 μm, the confinement effect of the microfiber on the light field significantly enhances, causing most of the light field energy to concentrate within the fiber core, while the evanescent field outside the fiber is relatively weak. This phenomenon suggests that as the diameter increases, more light energy is effectively confined within the core of the microfiber rather than escaping into the external space [20].

When the environmental medium surrounding a micro/nano optical fiber changes, the characteristics of its evanescent field will also change, significantly affecting the transmission of the optical field [21]. This property can be utilized to construct micro/nano optical fiber sensors based on evanescent field sensing [22]. The working principle is as follows: when the input optical signal is effectively coupled into the interior of the micro/nano optical fiber, part of the light propagates along the surface of the fiber in the form of an evanescent field within the sensing area. The energy carried by the evanescent field light exhibits extremely high sensitivity to changes in the physical and chemical parameters of the surrounding environment, thus achieving efficient sensing functionality. This energy perception mechanism can accurately capture variations in environmental parameters, leading to corresponding adjustments in the output optical signal of the sensor. By modulating the output signal at the sensor’s output terminal, it is possible to accurately obtain information related to changes in the parameters to be measured, thereby realizing the sensing function [23].

**Figure 2:**
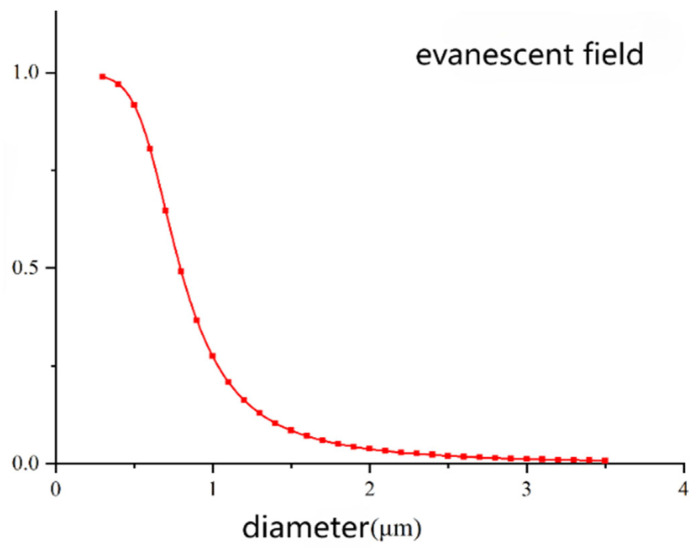
Variation in evanescent field ratio with diameter.

As shown in Figure 2, when the diameter of the microfiber is 300 nm, the proportion of the evanescent field reaches as high as 98.9%. As the diameter increases from 300 nm to 1 μm, there is a significantly decreasing trend in the proportion of the evanescent field. When the diameter exceeds 1 μm, the change in the proportion of the evanescent field becomes gradual and approaches 0. Based on the analysis of the relationship between the light field distribution of microfibers and their diameter, it can be inferred that using microfibers with a diameter smaller than the incident wavelength can achieve stronger evanescent field energy in practical sensing processes.

### 2.2. Multi-Mode Microfiber Interferometer

The multi-mode microfiber fabricated using the non-adiabatic tapering technique is a structure that can support the simultaneous transmission of multiple modes. This method is faster than traditional adiabatic techniques, resulting in a larger taper angle. When the input light wave propagates into the tapered region, its structural characteristics begin to take effect. The abrupt change in fiber diameter in this area effectively excites higher-order modes, leading to coupling and interference effects between the fundamental mode and the higher-order modes [24].

**Figure 3:**
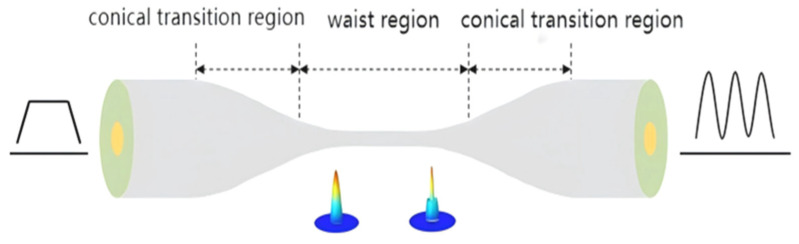
Multi-mode microfiber pattern interferometer.

The tapered microfiber structure consists of a standard single-mode fiber region, a tapered transition region, and a waist region. When an incident light wave reaches the first tapered region during transmission, a sudden change in the wave-guide structure induces the occurrence of higher-order modes. Consequently, the fundamental mode and excited higher-order modes propagate together until they reach the second tapered region, where coupling occurs again, allowing them to continue propagating. Throughout the entire light wave transmission process, transmission loss is minimal. Its structural diagram is shown in Figure 3.

In fiber transmission, the fundamental mode and other higher-order modes exhibit a certain optical path difference due to differences in their effective refractive indices. This optical path difference can lead to interference phenomena [25]. By utilizing the mode interference effect in the tapered transition region of microfibers, it is possible to successfully construct interference-type microfiber sensors. Additionally, evanescent waves in microfibers serve as a powerful tool [26], responding rapidly and effectively to subtle changes in external media, thus providing a solid foundation for creating high-sensitivity sensors. The diameter of multi-mode microfibers is generally less than 12 μm, primarily transmitting HE_11_ and HE_12_ modes. Currently, most microfibers used for sensing fall within this diameter range [27].

**Figure 4:**
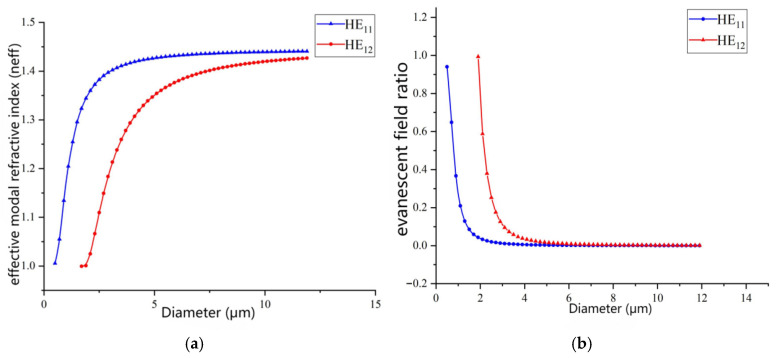
(**a**) Variation in effective refractive index with diameter for HE_11_ and HE_12_ modes in microfibers. (**b**) Variation in HE_11_ and HE_12_ mode abrupt field ratio with diameter in micro/nano optical fiber.

Research was conducted on the HE_11_ mode and HE_12_ mode. During mode analysis, the incident wavelength and fiber material were set, with a commonly used wavelength of 1550 nm, and the core refractive index was set at 1.4438, assuming the cladding is air. Study calculations were performed with a parametric scan of the fiber diameter, yielding effective refractive index data for both modes at different diameters, as shown in Figure 4a. As the fiber diameter increases, the effective refractive indices of both HE_11_ and HE_12_ modes also show an increasing trend. This change indicates that the light field confinement ability within the fiber is gradually strengthening, with more light being restricted inside the fiber. Notably, compared to the HE_11_ mode, the effective refractive index of the HE_12_ mode is smaller at the same diameter, suggesting that the HE_12_ mode is more sensitive to external refractive index changes.

The ratio of the evanescent field for both HE_11_ and HE_12_ modes was also calculated. As shown in Figure 4b, when the fiber diameter is small, the spatial distribution of the light field inside the fiber is relatively broad due to dimensional constraints, resulting in a significant amount of energy distributed in the evanescent field. Therefore, with smaller fiber diameters, the evanescent field energy can account for over 80%, indicating a stronger interaction between the light field and the external environment, providing a rich information source for sensing applications. However, as the core diameter gradually increases, the fiber’s ability to confine the light field also improves, leading to more stable propagation and a rapid decrease in the evanescent field energy proportion. When both HE_11_ and HE_12_ modes are present, the HE_11_ mode typically has a larger evanescent field proportion. This is due to the higher order of the HE_12_ mode, which includes more higher-order components. In sensing applications, this means that fiber sensors utilizing the HE_12_ mode are more sensitive to external changes, offering higher detection accuracy and a wider detection range.

## 3. Study on the Ultrasonic Sensing Characteristics of Multi-Mode Microfiber Interferometers

### 3.1. Research on Ultrasonic Field Propagation Characteristics Based on Multimodal Microfiber Sensors

#### 3.1.1. Model Development

**Figure 5:**
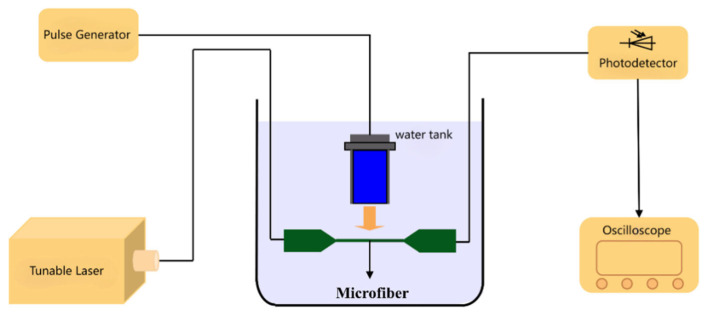
Microfiber mode interferometer ultrasonic sensing device.

Figure 5 shows the designed sensing experimental device, which efficiently converts acoustic signals into electrical signals. A tunable laser is used as an input light wave, set to an output wavelength of 1500 nm. The sensor’s output is connected to a photodetector and an oscilloscope, allowing for rapid and accurate conversion of light signals into electrical signals for precise measurement and analysis via the oscilloscope.

To ensure that acoustic wave energy fully interacts with the fiber, an ultrasonic pulse generator probe is positioned directly above the sensor. Additionally, to minimize divergence during propagation, the distance from the generation to the microfiber is kept within 2 cm. This ensures that ultrasound can fully interact with the evanescent field region of the fiber, enhancing the response speed and accuracy of sensing. To minimize interference from acoustic reflections, efficient acoustic-absorbing rubber is installed on the inner walls of the water tank. This rubber material effectively absorbs acoustic energy, preventing unnecessary reflections and disturbances within the tank.

**Figure 6:**
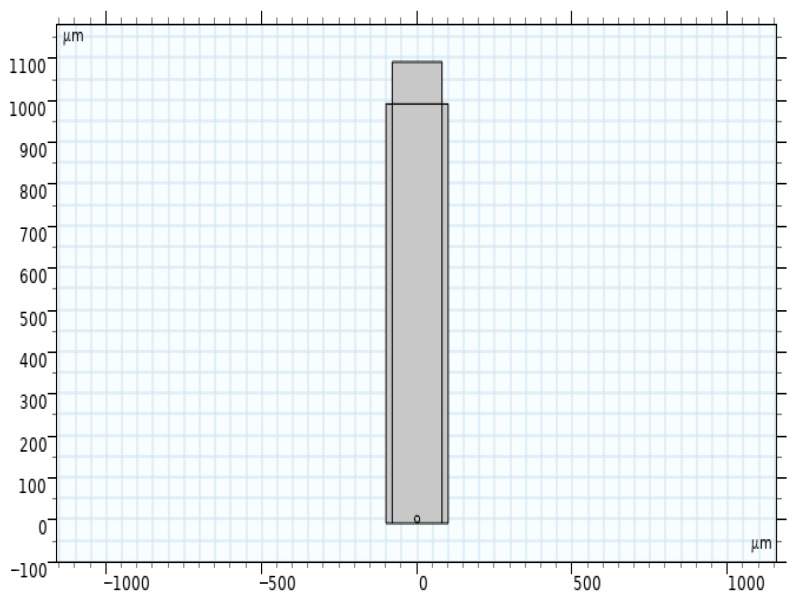
Two-dimensional cross-section propagation diagram of a model sensing device.

Using COMSOL to study the propagation of ultrasonic waves in a device, start by drawing the device’s 2D cross-section and establishing model parameters as shown in Table 1 to observe acoustic wave transmission from a 2D perspective. The results of the 2D model are illustrated in Figure 6.

To study an environment where the inner walls of a water tank are lined with acoustic-absorbing rubber, perfect matched layers (PML) are added to both sides of the model. The input light wave for the model is a tunable laser outputting at 1550 nm, and the model uses a piezoelectric sheet (5 MHz) as an acoustic source to study the ultrasonic pulse generator. The excitation function for the pulse signal is as follows [28]:

(2) f_n (t)=0.5(1−cos(2πft/5)) sin(2πft) (0<t<5/f)

#### 3.1.2. Finite Element Analysis of Ultrasonic Propagation

In 1943, Courant first introduced the concept of the Finite Element Method (FEM). The core idea of this method is based on variational principles [29]; however, for various reasons, it did not undergo sustained development at that time. It was not until the 1960s, over a decade later, that Clough formally brought the Finite Element Method to the forefront of research [30].

**Figure 7:**
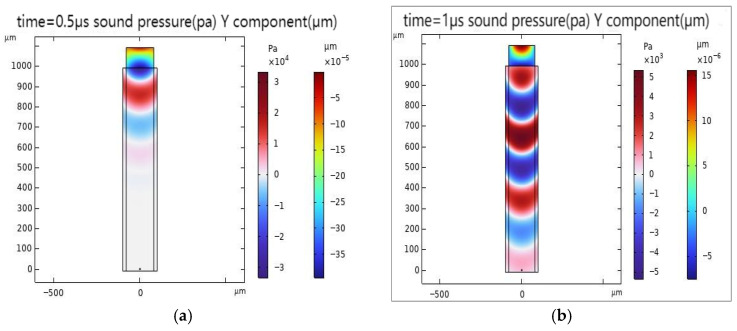
Modeled acoustic and electric field distributions for different time periods. (**a**) Distribution of the acoustic field and electric field in the model at 0.5 μs. (**b**) Distribution of the acoustic field and electric field in the model at 1 μs.

When performing transient analysis, the focus is mainly on the dynamic response of the model at different time points. Figure 7 shows the distribution of the acoustic field and light field when the acoustic wave has propagated for 0.5 μs and 1 μs. From Figure 7, it can be seen that the propagation of the acoustic wave within the model is relatively uniform, indicating that the acoustic design of the model is effective and can ensure stable forward transmission of the acoustic wave. This uniformity is crucial for applications such as acoustic wave detection, imaging, and communication, ensuring that the acoustic wave does not experience distortion or scattering during transmission due to inhomogeneities.

**Figure 8:**
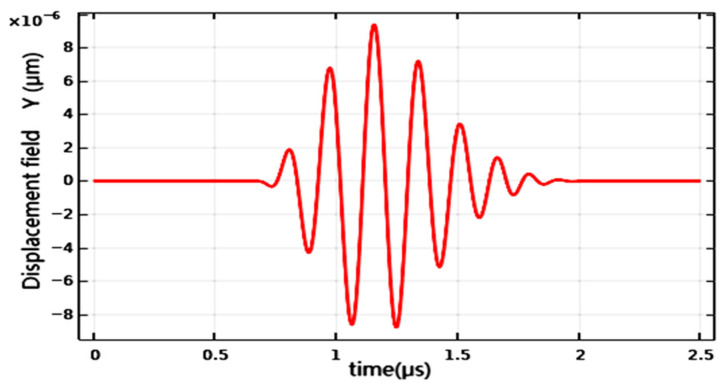
Acoustic waves detected by probes.

In delving into the results of acoustic wave transmission, as shown in Figure 8, it is clear that the acoustic wave successfully reached a designated detection point at approximately 0.7 μs. Further analysis reveals that the waveform at the detection point closely resembles the initial waveform. This observation is crucial for evaluating the effectiveness of acoustic wave propagation. Ideally, acoustic waves should maintain their original waveform during transmission to accurately reflect information from the source at the detection point, and the propagation effect displayed in Figure 8 undoubtedly aligns with this ideal state. Additionally, this favorable propagation characteristic is evidently stable and continuous throughout the process; Figure 8 shows no significant attenuation or distortion, indicating that the acoustic wave can efficiently carry information from the source to the detection point.

### 3.2. Experimental Study of Light Field Sensing Based on Multi-Mode Microfiber Sensors

#### 3.2.1. Theoretical Research

The evanescent field on the surface of the microfiber spreads into water. When acoustic pressure is applied from above, the refractive index of the intermediate medium (water) changes, leading to variations in the transverse mode field of the sensor and the corresponding effective refractive index. When ultrasound is applied in the sensing device, the relationship between the refractive index of water and the acoustic pressure can be expressed as [31]:

(3) $$n_{w}(p)=1+(n_{w,0}-1)(1+\frac{p-p_{0}}{p_{0}+Q}{)}^{\frac{1}{r}}$$

In the formula, sound pressure is denoted by the symbol p, and the Taylor coefficients r, $p_{0}$, and Q are represented by 7.44, 100 kilopascals (kPa), and 295.5 megapascals (MPa), respectively. Additionally, the refractive index of water under static conditions is expressed as 1.33.

When establishing the model, the optical parameters, acoustic parameters, and material parameters are set as shown in Table 2, Table 3 and Table 4. The experimental study of the acoustic pressure generated by the pulse generator is 100 kPa, and the expression for the excitation function is as follows [32]:

(4) $$V_{0}(t)=100\times e^{-(\frac{{\left(t-5T_{0}\right)}^{2}}{T_{0}})(\sin(2\pi f_{a}t))}$$

**Figure 9:**
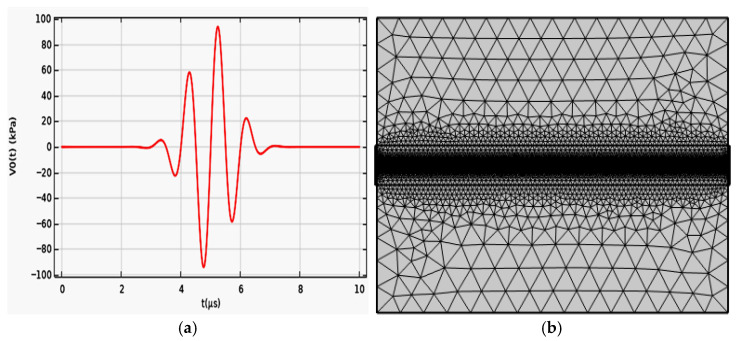
(**a**) 1 MHz excitation pulse signal. (**b**) Grid section of the experimental model of light field sensing.

In the formula, the frequency $f_{a}$ is set at 1 MHz, with t representing the time variable and $T_{0}$ denoting the driving signal period. The frequency domain waveform is illustrated in Figure 9a.

**Table 2:** Optical parameters.

Parameter Name	Expression	Value	Description
D0	2 [μm]	2 × 10^−6^ m	core diameter
D	5 [μm]	5 × 10^−6^ m	cladding diameter
L	200 [μm]	2 × 10^−4^ m	sensing region length
pml	1 [μm]	1 × 10^−6^ m	perfectly matched layer
Lda0	1550 [nm]	1.5 × 10^−6^ m	incident wavelength
f0	c_const/lda0	1.9986 × 10^141^/s	mode analysis frequency
air	150 [μm]	1.5 × 10^−4^ m	air domain
Ds	20 [μm]	2 × 10^−5^ m	water domain

**Table 3:** Acoustical parameters.

Parameter Name	Expression	Value	Description
distance	20 [μm]	2 × 10^−5^ m	distance from the acoustic source to the center
width	50 [μm]	5 × 10^−5^ m	source width
fa	1000 [kHz]	1 × 10^6^ Hz	pulse frequency
t	4.76 [us]	4.76 × 10^−6^ s	time
T0	1/fa	1 × 10^−6^ s	signal period
pressure	10 [kPa]	10 [kPa]	pressure
distance	20 [μm]	2 × 10^−5^ m	distance from the acoustic source to the center

**Table 4:** Material parameters.

Parameter Name	Expression	Value	Description
n_core	1.4438	1.4438	core refractive index
n_clad	1.43	1.43	cladding refractive index
n_w	1.3328	1.3328	refractive index of water under static conditions
rs	7.44	7.44	Taylor coefficient
p0	100 [kPa]	1 × 10^5^ Pa	Taylor coefficient
Q	295.5 [MPa]	2.955 × 10^8^ Pa	Taylor coefficient

The model was meshed using a free triangular grid, with a custom mesh set specifically for the optical fiber sensing area. During meshing of the sensing domain, the core domain cell size is set to lda0/5/n_core, the maximum cell size for the cladding domain is set to lda0/4/n_clad, and the minimum cell size is set to lda0/5/n_core. The maximum growth rate of the cells is 1.3, the curvature factor is 0.3, and the resolution for narrow regions is 1.

#### 3.2.2. Experimental Research

At a specific moment of 5.5 μs, the distribution of the acoustic field and electric field was observed, as shown in Figure 9b. It can be clearly seen from Figure 9 that the propagation of light in the core is not entirely uniform; some of the transmitted light spills out of the core.

**Figure 10:**
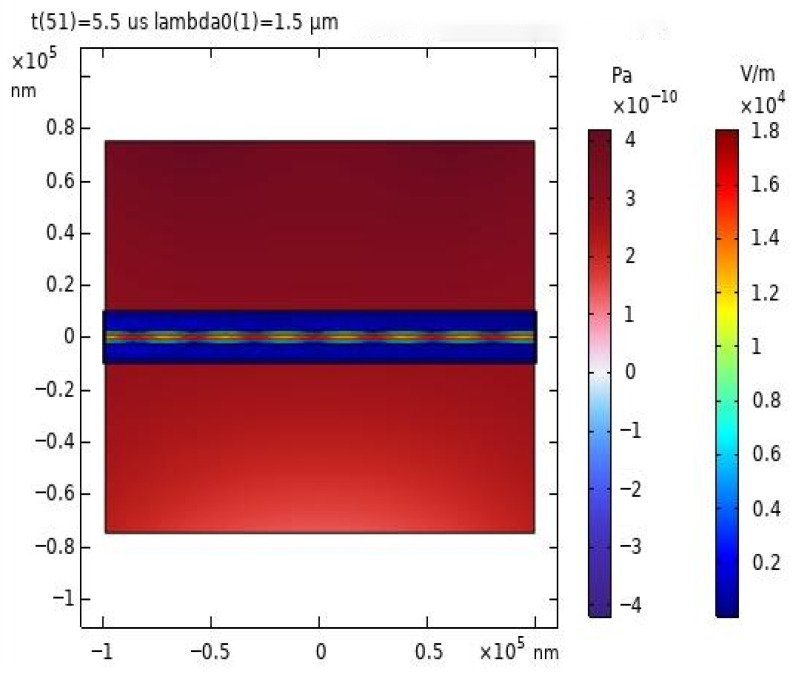
Modeled acoustic and electric field distributions at 5.5 μs in microfiber sensors.

In the in-depth study and analysis of the detection effects of microfiber sensors on acoustic waves, a key step is to calculate the total transmittance variation at the receiving port set in the model. Transmittance refers to the ratio of output light intensity to input light intensity as light passes through a medium, directly reflecting losses during transmission. For microfiber sensors, variations in transmittance are one of the important indicators for evaluating their performance. In the experiment, precise calculations of the total transmittance at the receiving port yielded changes in transmitted light power. The calculation process not only considers the material properties, structural design, and manufacturing processes of the fiber itself but also accounts for various effects generated during the interaction between acoustic waves and the fiber. The curve of transmitted light power variation visually illustrates dynamic changes in light power within the fiber under the influence of acoustic waves. From Figure 10, it is evident that as the acoustic wave signal changes, the total transmittance at the receiving port also varies, leading to fluctuations in transmitted light intensity. These fluctuations reflect the impact of acoustic wave signals on light transmission in the fiber, providing an important basis for evaluating the performance of microfiber sensors.

**Figure 11:**
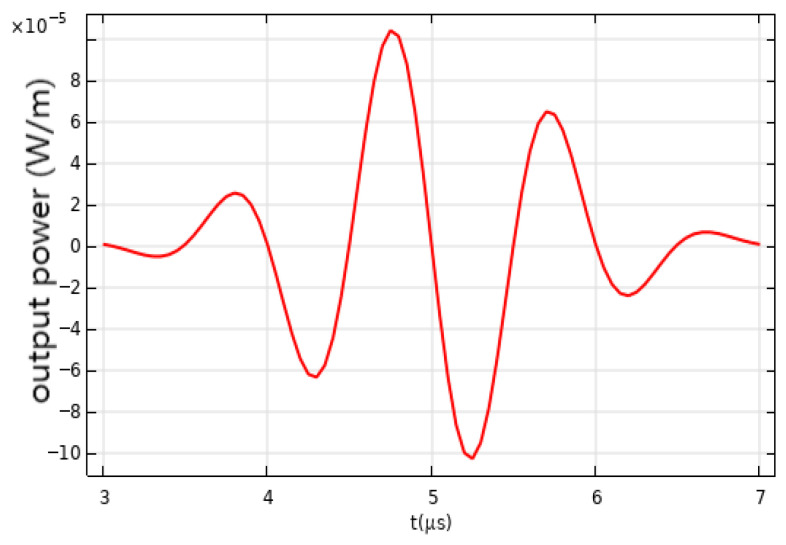
Optical power variation at the receiving port for 1 MHz pulse signals.

**Figure 12:**
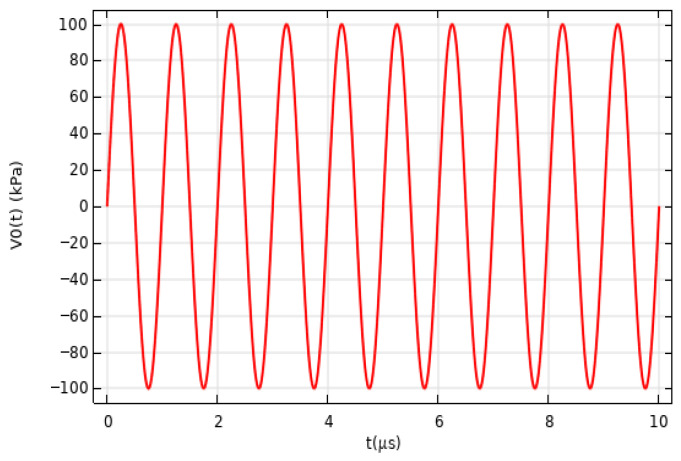
1 MHz continuous ultrasound signal.

When carefully comparing Figure 11 and Figure 12, it is clear that the detected signal waveform closely matches the original acoustic wave waveform. This result strongly demonstrates the outstanding performance of the proposed microfiber sensor in acoustic wave detection. To further validate the sensor’s performance, experiments were conducted to study the detection of continuous ultrasonic signals. In these experiments, continuous pulse signals with specific functional expressions were used as input, characterized clearly in both time and frequency domains. The response of the microfiber sensor to these input signals was then observed, with the function expression of the continuous pulse signal being [33]:

(5) $$V_{0}(t)=100\times \sin(2\pi f_{a}t)$$

The ultrasonic signal diagram is shown in Figure 12.

**Figure 13:**
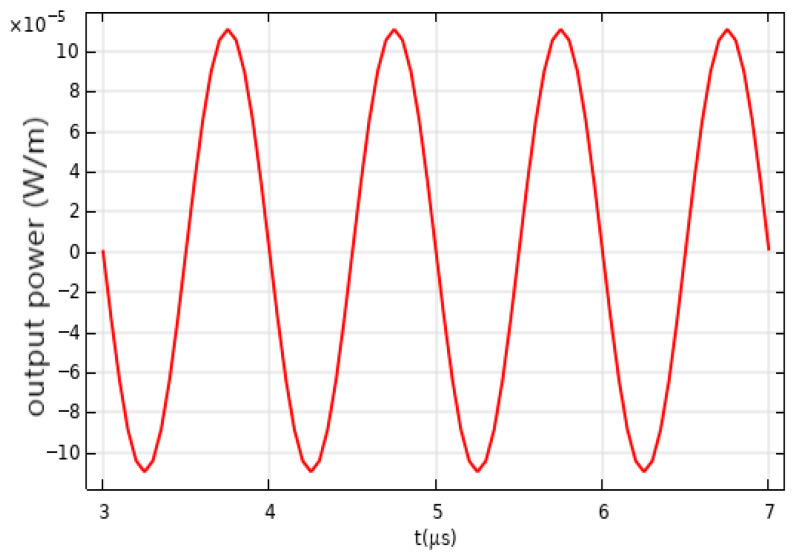
1 MHz continuous signal receiving port optical power variation.

In the display of Figure 13, comparing the original pulse signal with the detected signal waveform, it can be seen that both maintain almost identical waveform characteristics. This high degree of similarity not only reflects the sensor’s precise capture of acoustic wave signals but also highlights its efficiency and stability in the signal processing and conversion process, indicating that the sensor introduces almost no distortion or noise during acoustic wave detection. This high fidelity is crucial for acoustic wave detection, as it directly relates to the ability to accurately obtain and analyze useful information within the acoustic wave signals.

### 3.3. Comparison Experiment of Ultrasound Sensing Between Two Types of Fiber Optic Sensors

**Figure 14:**
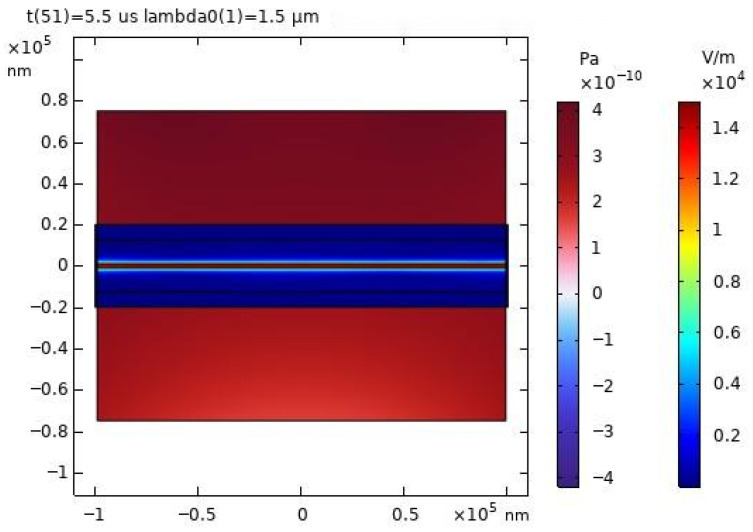
Modeled acoustic and electric field distributions at 5.5 μs in single-mode fiber sensors.

To accurately assess performance differences in different sensors in detecting ultrasonic waves, a modeling study was conducted under the same experimental conditions to investigate the detection process of a standard single-mode fiber optic sensor for ultrasonic waves. During this process, not only were the sensor’s response characteristics considered, but special attention was also paid to the distribution of acoustic and electric fields, as these are key factors in evaluating sensor performance. Figure 14 visually illustrates the distribution of acoustic and electric fields. First, it is observed that when incident light propagates within the core of a single-mode fiber, it exhibits a uniform characteristic. This uniformity indicates that the propagation path of light inside the fiber is stable and continuous, with no significant scattering or energy loss. This is an important feature of single-mode fibers, ensuring that optical signals can be transmitted efficiently and without distortion to the other end of the fiber. However, due to this uniform propagation characteristic, incident light does not form a significant evanescent field at the fiber surface. The evanescent field is a special phenomenon that occurs near the surface of the fiber, highly sensitive to minute changes in the external environment. In ultrasonic wave detection, the presence of an evanescent field can greatly enhance the sensor’s responsiveness to ultrasonic signals [34]. Unfortunately, standard single-mode fibers, due to their specific structural and material properties, struggle to create a significant evanescent field at their surface [35].

**Figure 15:**
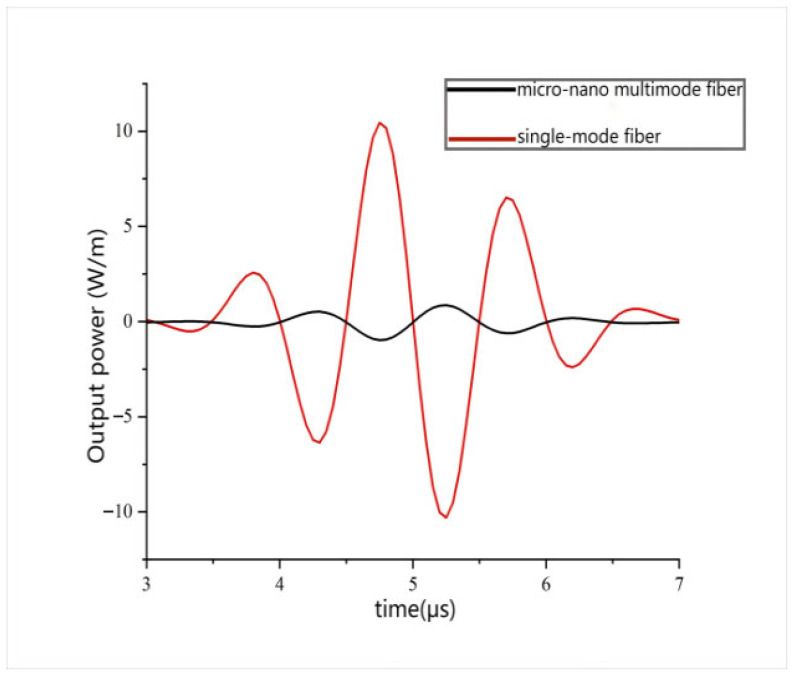
Output optical power of acoustic sensors of single-mode fiber and multi-mode microfiber.

Simultaneously, changes in the total transmittance at the receiving port of a standard single-mode optical fiber sensor were calculated, and a transmission optical power diagram was drawn based on this data. In Figure 15, the waveform results of output power detection from two sensors are clearly compared. From the comparison results, significant performance differences between the two can be intuitively observed. The output amplitude of the single-mode optical fiber sensor is noticeably lower, showing a significant deficiency in its response capability compared to the multi-mode microfiber sensor. This result directly reflects the limitations of the single-mode optical fiber sensor in ultrasonic detection. Due to its unique structural design and material properties, light in a single-mode fiber propagates along a relatively fixed path, making it difficult to form a strong evanescent field effect. When ultrasonic waves act on the fiber, a weak evanescent field results in a relatively weak interaction between the fiber and ultrasonic waves, limiting the sensor’s response capability to ultrasonic waves. In contrast, the multi-mode microfiber sensor exhibits outstanding performance. The internal multi-mode light propagation allows the fiber surface to create a strong evanescent field effect. When ultrasonic waves act on the fiber, this evanescent field interacts strongly with ultrasonic waves, significantly enhancing the sensor’s response capability. Therefore, in practical ultrasonic detection applications, the multi-mode microfiber sensor can more accurately capture ultrasonic signals and convert them into observable output signals.

**Figure 16:**
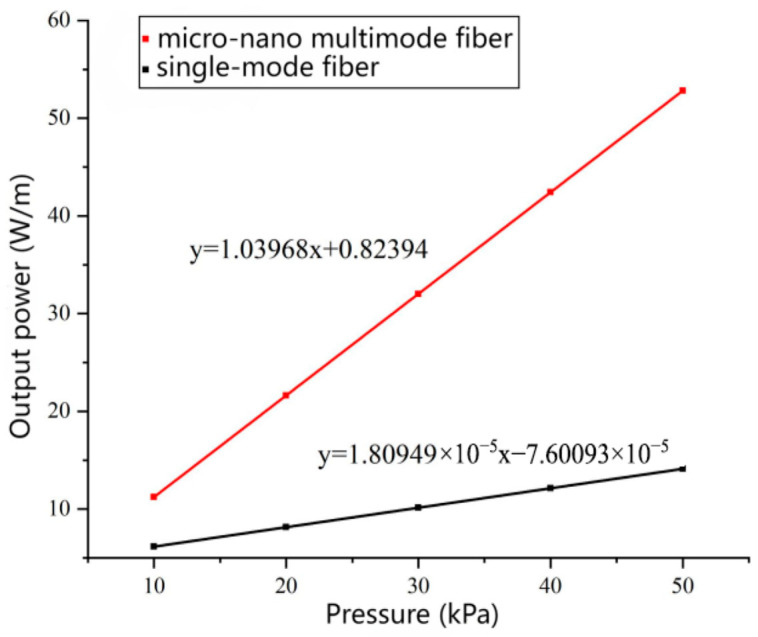
10 kPa~50 kPa single-mode and multi-mode microfiber acoustic sensor output optical power.

Through computational analysis, this study thoroughly investigates the output power peak performance of two types of sensors within an acoustic pressure range of 10 kPa to 50 kPa. To visually present the performance differences between the two sensors, the obtained data is plotted as shown in Figure 16. A curve fitting method was employed to process the data. The fitted linear equations approximate the sensitivity of the two sensors, represented by the slopes of the lines. The results are shown in Table 5, where the slope of the standard single-mode optical fiber sensor is relatively low, while the slope of the microfiber sensor is significantly higher at 1.03968. This sensitivity difference mainly arises from variations in material structure and response mechanisms between the two sensors. The single-mode optical fiber sensor, due to the inherent rigidity of its material medium, exhibits relatively low sensitivity when responding to ultrasonic acoustic pressure. When ultrasonic acoustic pressure acts on the single-mode fiber, the internal medium changes very little, making it difficult to generate sufficient signal output. In contrast, the microfiber sensor demonstrates outstanding high sensitivity characteristics. When ultrasonic acoustic pressure is applied to the microfiber, it causes significant changes in the intermediate medium. This alteration not only affects the internal structure of the microfiber but also leads to strong interactions with the evanescent field generated at the surface of the microfiber. The evanescent field is a special phenomenon where light waves exist near the surface of the fiber, making it very sensitive to small changes in the external environment. Therefore, when ultrasonic acoustic pressure acts on the microfiber, changes in the surface evanescent field are rapidly amplified and converted into strong signal outputs, significantly enhancing the sensor’s response capability.

The microfiber sensor demonstrates significant advantages in biomedical photoacoustic imaging technology. Its high sensitivity enables it to capture ultrasonic signals more accurately and convert them into observable output signals, thereby enhancing imaging precision and reliability.

### 3.4. Research on the Acoustic Detection Characteristics of Sensors

**Figure 17:**
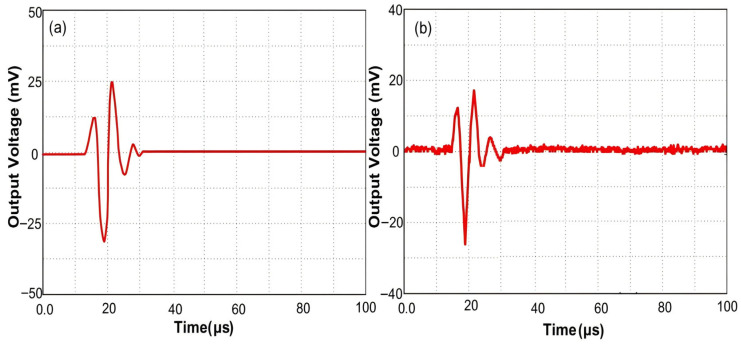
(**a**) Comparison of detection waveforms between single-mode fiber and micro/nano fiber sensors. (**b**) Graph showing the relationship between measured output voltage and sound pressure [21].

Figure 17a presents the single-pulse sound pressure waveform generated by the pulse generator, showing a peak amplitude of 25 mV and a lowest voltage of −31.25 mV. The amplitude difference corresponds to a sound pressure value of 10.03 kPa. Figure 17b displays the electrical signal obtained after conversion, with a measured peak amplitude of 17.43 mV and a trough amplitude of −26.77 mV. Figure 17 indicates that the detected signal closely resembles the original signal waveform. The inset in Figure 17b reveals that the pulse repetition frequency of the detected acoustic signal is 5 kHz, with background noise primarily caused by the electrical noise of the photodetector (PD). The noise level, approximately 0.1 mV, corresponds to a pressure of 0.54 kPa, representing the minimum detectable sound pressure value. Increasing the incident power of the tunable laser and observing the background noise with a spectrum analyzer did not yield significant improvements, suggesting that photon shot noise is not the dominant factor in the background noise. Compared to the original waveform, the detected signal profile has some minor irregularities due to the applied pulse acoustic wave not being a perfect plane wave. Given the high stiffness of quartz glass and the applied sound pressure of only kilopascals, the resulting phase change is negligible, making it an unlikely cause of distortion. It is important to note that temperature-induced changes in interference fringe drift, at 8 pm/°C, could significantly affect sensing sensitivity. However, since the experiment was conducted at room temperature in water, temperature effects can be disregarded. Therefore, by positioning the incident laser wavelength near the maximum slope of the interference peak, we can achieve a micro/nano fiber sensor with enhanced sensitivity and stability.

### 3.5. Experiment on the Frequency Response Characteristics of Sensors

**Figure 18:**
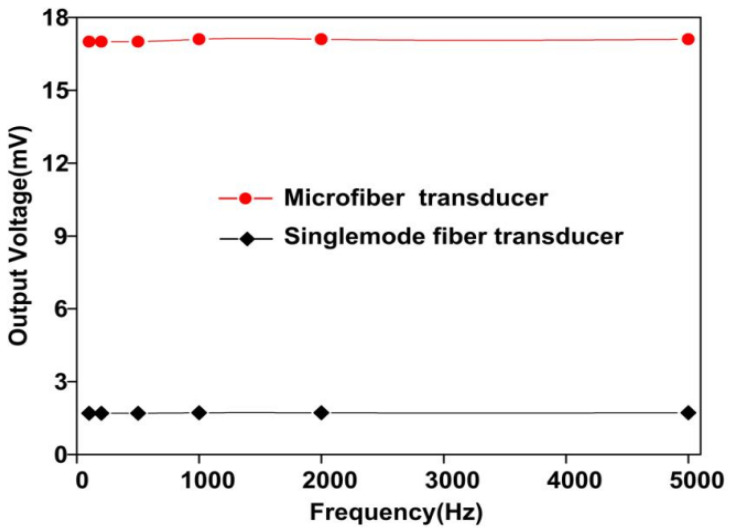
Frequency response of acoustic sensors made of single-mode fiber and micro/nano fiber when subjected to a sound pressure of 10.03 kPa [21].

Figure 18 illustrates the frequency response of acoustic sensors constructed from micro/nano fiber and single-mode fiber, spanning a range from 100 Hz to 5 kHz. Both sensors demonstrate a consistent response across this range, characterized by a flat frequency response curve, indicating their stability and superior performance.

## 4. Conclusions and Future Work

The paper proposes a structure of a microfiber sensor based on mode interference and its detection of ultrasound:

(1) The simplified Helmholtz equation is used to construct a model of a microfiber with a step-index distribution of refractive index, and the analysis of the coupling theory of the fundamental mode and higher-order modes in the microfiber is carried out. The model of the microfiber is established using finite element analysis software, and the optical physical field is set. The light field distribution inside the microfiber is calculated under different sizes, incident wavelengths, and the refractive properties of the surrounding environment, and the change in the proportion of the evanescent field on the surface of the fiber is calculated.
(2) According to the characteristics of multi-mode microfiber mode distribution, a mode interference microfiber sensor structure based on HE_11_ and HE_12_ modes is proposed. The effective refractive index and the proportion of the evanescent field of the HE_11_ and HE_12_ modes are investigated in detail by theoretical analysis. In addition, the effect of the group effective refractive index difference on the sensitivity in the process of refractive index and temperature measurements is analyzed; the refractive index sensitivity of a microfiber SPR sensor in the same environmental medium is further compared.
(3) The diameter of the microfiber is usually less than 10 μm, and the whole core is relatively uniform; the microfiber sensor studied is applied to ultrasound detection. By setting the acoustic physical field of the model in solid and liquid environments, the transmission characteristics of ultrasound are simulated. Thus, the model of an ultrasound-sensing experimental device and related physical field settings are constructed. The detection effects of the multi-mode microfiber interferometer and an ordinary single-mode fiber sensor under the action of ultrasound are compared experimentally; it was found that the output light intensity of the multi-mode microfiber interferometer maintains the original waveform and the sensing sensitivity is much higher than that of the ordinary single-mode fiber sensor, which has an impact on the quality of optical and acoustic signal reception.

## Figures and Tables

**Table 1:** Parameter setting.

Parameter Name	Expression	Value	Description
fa	5 [MHz]	5 × 10^6^ Hz	pulse frequency
d_core	6 [um]	6 × 10^−6^ m	core diameter
d_clad	20 [um]	2 × 10^−5^ m	cladding diameter
L	1550 [nm]	1.55 × 10^−6^ m	Incident wavelength
rho	7500 [kg/m^3^]	1.9986 × 10^141^/s	density of piezoelectric material
n1	1.4438	1.4438	core refractive index
n2	1.3328	1.3328	refractive index of water

**Table 5:** Ultrasonic sensitivity of two fiber optic transducers.

Optical Fiber Sensor	Acoustic Pressure	Sensitivity
Multi-mode microfiber sensor	100 kPa	1.03968 W/mkPa
Single-mode optical fiber sensor	100 kPa	1.809491 × 10^−5^ W/mkPa

## Data Availability

The original contributions presented in this study are included in the article. Further inquiries can be directed to the corresponding author.
